# The Importance of Research on Occupational Sedentary Behaviour and Activity Right Now

**DOI:** 10.3390/ijerph192315816

**Published:** 2022-11-28

**Authors:** Bronwyn K. Clark, Charlotte L. Brakenridge, Genevieve N. Healy

**Affiliations:** 1 School of Human Movement and Nutrition Sciences, The University of Queensland, St Lucia 4072, Australia; 2 Baker Heart and Diabetes Institute, Melbourne 3004, Australia; 3 School of Physiotherapy and Exercise Science, Curtin University, Perth 6845, Australia

The workplace has been identified as a key setting for public health interventions to ‘promote and maintain the highest degree of physical, mental and social well-being of workers in all occupations’ by the World Health Organisation [1]. Work time forms a large portion of the day, and healthy and non-healthy behaviours performed during this time can have a significant impact on health. For example, long periods of sitting at work [2] or large proportions of work time spent in moderate-to-vigorous physical activity [3] have been identified as detrimental to health, whereas interrupted sitting and time spent undertaking light-intensity activity at work can be beneficial [4,5]. As workplaces are becoming increasingly less active [6] and technology advances are resulting in the automation and computerisation of many occupations [7], we can expect that work will demand less movement and allow more sitting time, making this an increasingly important target for workplace health.

Over the last 10 years, a number of interventions targeting the health of workers have concentrated on breaking up prolonged sitting in high-sitting and low-activity workplace environments [8]. Many successful interventions have addressed multiple levels of influence on behaviours, including at social, ecological, individual, environmental, and policy levels [9,10]. For example, perceptions of manager and colleague support for activity have been associated with reductions in workplace sitting time [11], and environmental changes such as installing sit–stand desks and active building design have demonstrated benefits for sitting reduction and increased movement [12,13,14]. Despite this evidence, workplaces still vary widely in their activity-supportive characteristics, with a study in 230 organisations showing that nearly all had room for improvement [15]. Indeed, it has been argued that not all workplaces are providing a safe system of work in terms of addressing prolonged occupational sitting [16]. Employers have a responsibility for worker health—particularly in relation to health issues arising from doing the job. Therefore, there needs to be continued evaluation of the impact of these multiple influences on behaviour, and the extent to which there is the capability, motivation, and opportunity to change behaviour in workplaces [17].

Research into occupational sedentary behaviour and activity is extremely pertinent in the context of the COVID-19 pandemic. The pandemic resulted in large changes to workers’ physical and social work environments, which have important influences on workplace behaviour (including activity and sedentary behaviour) [18]. Both total PA and occupational PA decreased and sedentary time increased overall during the pandemic [18,19]. Furthermore, greater time working from home is associated with higher levels of sedentary time [20], with some workers increasing their daily sitting time by two hours or more [21]. While working from home gives more flexibility for workers to be active outside of work hours [22], this may not be enough to counter the decreased activity in other areas, such as at work and in active transport [23], or to counteract other barriers to activity resulting from working from home, such as prolonged working hours [24]. These findings highlight the importance of understanding how activity behaviour might vary across working from home, office and hybrid work patterns. There is also a need to explore active and sedentary behaviour patterns and interventions across different industries; much of the research undertaken during the pandemic was conducted in relation to office workers, university workers and professionals [18,25], with less known about specific industries that have high levels of sitting time, such as call centre workers.

Many of the work-related changes experienced in the COVID-19 period are expected to remain or change again. A study of professionals in office buildings in the real estate sector identified that the post-pandemic era is likely to involve flexible working with a hybrid approach between remote and office-based working [26], while polls from North America after work from home orders were lifted showed a preference for continued partial work from home [27]. Research has also identified that 36% of the jobs in the European Union are suitable for remote working, even after pandemic restrictions [28]. Thus, a priority for research going forward is understanding the impact of post-COVID-19 changes on how, where, and when we work, and the consequences for workers’ health and behaviours [26].

In terms of workplace health promotion, what worked before COVID-19 may no longer be feasible or effective outside of a traditional workplace. Programs that leverage face-to-face contact, physical products (e.g., workstations) or environmental features (e.g., stairs) may not work in a home environment. There needs to be a rethink in understanding and promoting workplace health with a hybrid workforce, as the ecological impacts on behaviour will likely be different at work to those at home. Those who work from home potentially have more flexibility in the way they work, but there may be more barriers to standing at work (e.g., not having a sit/stand desk) and there is less peer influence to change behaviour. One study (pre-COVID-19) on the implementation of a flexible work policy found there were no changes in physical activity after the introduction of flexible location working arrangements; however, sitting time increased both on days the employees worked at home and on days they worked at the office [29]. Reducing sedentary time at home can be effective in the short-term, using many of the same strategies that have been used previously, provided they can be brought home (e.g., allowing sit–stand desks to be brought home) or can be remotely delivered (e.g., web-based programs) [30,31]. However, it is unclear how these strategies work in the long term, and an added challenge will be addressing work behaviour taking into account the mix of work styles and environments likely to make up working hours.

In order to move forward post-COVID-19, it is clear we need to be flexible and use different approaches to promote occupational activity and reduce sitting time to take into account the environments in which work occurs. Targeting and evaluating interventions requires both accurate and context-appropriate measurement methods. We cannot assume previously validated office measures will work in work-from-home settings. For example, an established sitting questionnaire that has shown acceptable validity for use in office environments—the Occupational Sitting and Physical Activity Questionnaire—showed lower correlations with objective measures of sitting for work-from-home settings [32]. Adding to the considerations for measurement selection for researchers is the rapid increase in the technology available for measuring activity [33]. Technology-based methods of smartphone accelerometers [34], Fitbits [35] and research-grade accelerometers [36] were used to measure physical activity and sedentary behaviour during the pandemic and can be used across work and home environments. These provide higher accuracy than self-reports, but minimal information regarding context, which is necessary to target setting-specific changes in active and sedentary behaviour. Context can be gained using self-report data capture techniques such as online work diaries or momentary sampling [37], or through the use of additional devices such as workplace identification tags enabled for Bluetooth and radio frequency [38,39] or desk-height monitors for detecting sit–stand desk use [40]. These methods have not been tested in the home environment, so may need further development and adaptation for use in hybrid work or work-from-home settings.

Newer technologies may provide measurement solutions for multiple work environments. There is emerging evidence that researchers can take advantage of the existing wireless signals from mobile or smart devices (e.g., laptops, smartphones and smart electronics) to determine features such as daily activity and indoor localization [41]. These have been used in homes for energy need applications [42] and in traditional offices for detecting sitting and walking [43], suggesting such methods could be employed in work-from-home applications. There are many more developing technologies that can assess human behaviour, and multimodal combinations of these technologies provide considerable potential, though more development is required to ensure solutions are user friendly [44]. The challenge going forward will be harnessing the right technology to provide information in a usable way across the multiple environments where work takes place. It is likely that a combination of methods will work best, and that the methods may differ depending on workplace setting and research question.

In the context of changing workplace activities and environments in the post-pandemic setting, and the rapid expansion of technology available for work and for assessing behaviour at work, researchers must be flexible in how they plan and intervene with workplace health. Studies assessing changes to increase health-enhancing movement and reduce prolonged sitting time at work must be appropriate to the varied occupational environments in which work occurs, including hybrid work and working from home. Going forward, there is also a need to develop measures suitable for these differing work environments that provide accurate evidence for how, when and where activity and sedentary behaviour occur, and to evaluate interventions and workplace changes. Hybrid work has become the “new normal” and, as such, it is imperative that our evaluations, interventions, and measurements in workplace health promotion are in line with this change. As workplace health researchers, it is also important that we remain flexible and responsive to future changes and challenges in workplace health research.
